# ‘It’s far from the norm’: breastfeeding beyond 1 year in the Republic of Ireland

**DOI:** 10.1093/heapro/daae088

**Published:** 2024-08-17

**Authors:** Gillian Paul, Niamh Vickers, Regina Kincaid, Denise McGuinness

**Affiliations:** School of Nursing, Psychotherapy and Community Health, Dublin City University, Collins Ave Ext, Whitehall, Dublin 9, Ireland; School of Nursing, Midwifery and Health Systems, Health Sciences Centre, University College Dublin, Belfield, Dublin 4, Ireland; Independent IBCLC, Malahide, Co. Dublin, Ireland; School of Nursing, Midwifery and Health Systems, Health Sciences Centre, University College Dublin, Belfield, Dublin 4, Ireland

**Keywords:** breastfeeding, infant feeding, child health, maternal health, peer support, health promotion

## Abstract

Breastfeeding is the optimal form of nutrition for infants and young children. The World Health Organization recommends that babies are breastfed exclusively for the first 6 months of life, and up to the age of 2 years or beyond in combination with complementary food. Breastfeeding initiation and continuation rates are suboptimal globally and very low in the Republic of Ireland where health promotion initiatives and healthcare professional support predominantly focus on the important phase of initiation and early months of the breastfeeding journey. This qualitative descriptive study aimed to explore the experiences of women who chose to breastfeed their children beyond 1 year of age in the Republic of Ireland. Fourteen women participated in semi-structured interviews. Interviews were transcribed verbatim and thematic analysis was conducted. The analysis generated three overarching themes: (1) Influences on breastfeeding beyond 1 year, (2) Sustaining breastfeeding and (3) Benefits of breastfeeding beyond 1 year. Family, friends, peers, culture and commercial milk formula marketing had an influence on breastfeeding journeys. Support, determination, knowledge, bed-sharing and Covid-19 pandemic social restrictions helped to sustain breastfeeding beyond 1 year. Benefits of breastfeeding beyond 1 year such as nutrition, strengthening of emotional bonds, development of a parenting tool, and protection of child and maternal health were identified. Our findings support the need for discussions and further research on the normalization of breastfeeding beyond 1 year in the Republic of Ireland, targeted health promotion initiatives and education programmes for healthcare professionals on supporting the continuation of breastfeeding.

**Contribution to Health Promotion**

This paper, presenting the experiences of women who breastfeed beyond 1 year in the Republic of Ireland, raises several issues pertinent to health promotion including:

- The importance of enhancing health literacy of parents and the general population in relation to breastfeeding beyond 1 year.
- The need for education programmes for healthcare professionals on supporting the continuation of breastfeeding.
- The influence commercial companies have on breastfeeding practices and outcomes.
- The obligation of governments and communities to promote breastfeeding and to create a supportive culture and environment for breastfeeding dyads.

## BACKGROUND

The World Health Organization (WHO) (WHO, 2019) recommends that babies are breastfed exclusively for the first 6 months of life, and up to the age of 2 years or beyond in combination with complementary foods. Breastfeeding duration internationally remains significantly below this recommendation, with a global average of 8.7 months of any breastfeeding reported by the World Breastfeeding Trends Initiative (Zakarija-Grković *et al*., 2020).

Breastfeeding rates in the Republic of Ireland (RoI) are the lowest in Europe (Beatha and O’Sullivan, 2022) and among the lowest globally. Recent data show that on discharge from the maternity services, 37% of infants were exclusively breastfed and 21% were breastfed non-exclusively [National Women and Infants Programme (NWIP), 2023]. At 3 months, 35% of infants were reported to be breastfeeding, exclusively or non-exclusively (Theurich *et al*., 2019). There is no systematic monitoring of breastfeeding rates in the RoI beyond 3 months of age (Theurich *et al*., 2019). Consequently, there are no national data routinely collected hence it is not possible to calculate the duration of breastfeeding or the rates of breastfeeding at 1 year of age or beyond (World Breastfeeding Trends Initiative, 2023).

Health benefits of breastfeeding in children include protection from infections, reduced hospital admissions and a reduced risk of sudden infant death syndrome (SIDS) (Victora *et al*., 2016; Thompson *et al*., 2017; Murphy *et al*., 2023). Children who are breastfed are also likely to have a reduced risk of obesity (McCrory and Layte, 2012; Pluymen *et al*., 2019; Horta *et al*., 2023) and developing diabetes (Victora *et al*., 2016). In women, breastfeeding is associated with a reduced risk of breast and ovarian cancer, as well as cardiovascular disease (Chowdhury *et al*., 2015; Stordal, 2023).

There is growing evidence that a longer duration of breastfeeding results in further protective effects for the mother–child dyad. Human milk in the second postpartum year was identified as having higher concentrations of total protein, lactoferrin, lysozyme and immunoglobulin A (Perrin *et al*., 2017). The natural immunological properties of breastmilk increase as the young child encounters further pathogens in their environment (Pickett, 2022). Women who breastfed longer than 12 months have less incidence of diabetes, hypertension, hyperlipidaemia and cardiovascular disease than those who do not (Schwarz *et al.*, 2009; Peters *et al*., 2017). For every 12 months of breastfeeding, the risk of breast cancer is reduced in women by 4.3%, while women who breastfeed for 6–12 months have a 28% reduction in risk of developing ovarian cancer (Stordal, 2023). Breastfeeding can also benefit maternal mental health. When women reach their breastfeeding goals, there is a lower incidence of guilt and postpartum depression (Borra *et al*., 2015; Brown, 2016).

Breastfeeding is linked to several of the Sustainable Development Goals (WHO, 2020) and is recognized to be crucial to national development and planetary health (Perez- Escamilla, 2017). According to Rollins *et al*., ‘improvements in breastfeeding would help achieve the targets for health, food security, education, equity, development, and the environment’ (Rollins *et al*., 2016, p. 501). For example, an estimated 823 000 children’s lives could be saved each year in low- and middle-income countries if breastfeeding practices were at optimal levels (Victora *et al*., 2016).

Supporting breastfeeding for as long as mothers and their children wish requires intersectoral polices aimed at reducing barriers to breastfeeding and raising public awareness of its importance (Pérez-Escamilla *et al*., 2023). Central to this is addressing the commercial milk formula (CMF) industry’s violation of the WHO International Code of Marketing of Breastmilk Substitutes and further regulation of the industry, including ending the marketing of CMF (Rollins *et al*., 2023). Industry marketing reinforces and exacerbates misconceptions by offering CMF as a solution to ‘normal infant behaviours’, which in many cases results in unnecessary introduction of CMF and the cessation of breastfeeding earlier than planned (Pérez-Escamilla *et al*., 2023).

‘Breastfeeding beyond infancy’ (Brockway and Venturato, 2016), ‘breastfeeding beyond one year’ (Lackey *et al*., 2021), ‘extended breastfeeding’ (Baranowska *et al*., 2018), ‘natural term breastfeeding’ (Thompson *et al*., 2020), ‘prolonged breastfeeding’ (Caulfield *et al*., 1996) and ‘breastfeeding long-term’ (Dowling and Brown, 2013) are all terms used in the literature to describe the practice of the continuation of breastfeeding after the infant’s first birthday. Pickett (Pickett, 2022) is cautious in relation to terms used to describe the continuation of breastfeeding and offers the opinion that the use of the term ‘extended breastfeeding’ conveys meaning of ‘beyond the normal’. Cognizant of this ‘breastfeeding beyond 1 year’ is the term used in this study.

Previous qualitative research on breastfeeding beyond 1 year in the UK reports that women feel under pressure to breastfeed infants but are discouraged to continue breastfeeding as their children get older (Thompson *et al*., 2020). Similarly, Jackson and Hallam (2021) and Dowling and Brown (2013) describe in their research findings the cultural stigma women faced when they breastfed beyond infancy. These studies reinforce Dowling and Pontin’s (2017) which explored the experience of women breastfeeding their babies for over 6 months through a liminal lens. Their findings suggest breastfeeding beyond 1 year of age can place women in a liminal space whereby they feel they are incongruent to society’s views, to the extent that some women who continue to breastfeed despite societal pressure reported feeling isolated.

Cultural, religious and societal norms may contribute to low breastfeeding rates in the RoI (Philip *et al*., 2023). These include issues such as negative attitudes and social perceptions towards breastfeeding, personal embarrassment and family dynamics (Philip *et al*., 2023). Targeted interventions which take cognizance of context are required to normalize breastfeeding (Gallegos *et al*., 2020). Our research will contribute to the evidence base by providing context of the previously under reported experience of women who breastfeed their children beyond 1 year in the RoI.

## METHODS

### Aim

This aim of the study was to explore women’s experiences of breastfeeding beyond 1 year in the RoI.

### Research design

A qualitative descriptive research design within an approach of pragmatism (Allemang *et al.*, 2022) was deemed the most appropriate design to gain a broad insight into women’s experience of breastfeeding beyond 1 year in the RoI. The qualitative descriptive design was chosen as it is inductive, subjective and designed to develop an understanding of and describe the phenomenon (Bradshaw *et al*., 2017). This approach is particularly useful in providing first-hand insight and a rich description of an individual’s experience of a specific topic (Neergaard *et al*., 2009).

### Sample and recruitment

Inclusion criteria for participation in the study were: over 18 years of age, experience of breastfeeding a child for more than 1 year within the 2 years prior to interview, resident in the RoI and ability to participate in an interview in English. Purposive sampling was employed to deliberately select participants that met the inclusion criteria (Bowing, 2014). Participants were recruited via a Facebook page specifically related to supporting breastfeeding in the first year of life and beyond in the RoI. It is a private Facebook page with a very large membership, open to breastfeeding parents who are living or have lived in Ireland. The lead author contacted the administration team and provided them with the details of the study and confirmation that ethical approval was secured. The administrators agreed to post a message about the study on their Facebook page directing members to contact the lead author by email if they were interested in participating. The sample size of 14 participants was ascertained pragmatically according to concepts of information power and was influenced by the specific study aim, the relevance of participants’ experiences to breastfeeding beyond a year and the strong interview dialogues (Malterud *et al*., 2016).

### Data collection

Semi-structured interviews were conducted to elicit rich information on the participants’ experiences of breastfeeding beyond 1 year. An interview guide (Supplementary File 1) was developed reflecting the study aim and piloted with a breastfeeding mother. It was refined following this pilot interview. The interviews were conducted by the authors, all of whom have experience of supporting breastfeeding mothers as a midwife, and in three cases also as an International Board Certified Lactation Consultant (IBCLC). Each author interviewed at least one participant and discussed the interview with the lead author. Interviews lasted approximately 45 minutes to 1 hour and were held over the communications platform Zoom. At the end of the interviews, a summary of the content was given to the participants, and they were asked if they agreed with it and if they had anything further to add. The interviews were conducted in early 2022 as the public health restrictions for the Covid-19 pandemic were being lifted in the RoI.

### Data analysis

Interviews were transcribed verbatim. Each author transcribed the interviews they conducted. NVIVO software 12.1 was used for storing, organizing and coding the data. Thematic analysis, commonly used in qualitative descriptive research (Doyle *et al*., 2020), was led by a (G.P.) according to Braun and Clarke’s six phases of reflexive thematic analysis (Braun and Clarke, 2022). The transcriptions were read and re-read by two authors (G.P. and D.McG.). The data were coded, with codes relating to similar issues categorized into initial themes. These initial themes were reviewed by the two authors who, following discussions, subsequently refined the analysis by defining clear demarcated theme and subtheme headings. The results are presented below as a narrative, supported by pseudo-anonymized extracts from the data with the aim of illustrating the key findings.

### Ethical considerations

Ethical approval was granted from the Dublin City University Research Ethics Committee. On expressing interest in the study, potential participants were forwarded the Participant Information Leaflet outlining the study in detail including data collection, storage and protection procedures, and their right to withdraw from the study. They were invited to contact the lead author if they had any questions. Participants gave written informed consent prior to being interviewed and verbal consent at the commencement of the interview. The transcripts were pseudo-anonymized prior to analysis.

The study is reported according to the Standards for Reporting Qualitative Research (SRQR) devised by O’Brien *et al*. (O’Brien *et al*., 2014).

## RESULTS

Fourteen women who had breastfed a child for over 1 year in the previous 2 years were interviewed. The women all identified as Irish and resident in the RoI. They ranged in age from 30 to 44 years. All the women were in a heterosexual relationship and were married or cohabiting with their partner. Ten lived in urban settings and four in rural settings. All were educated to third level, having attended a university or another higher education institution. Two participants were first-time mothers, and 12 had more than one child. There was varying experience of breastfeeding among the women from breastfeeding one child to breastfeeding five children. Two participants were breastfeeding twins, and four participants were tandem feeding at the time of the interview.

Three core themes each with sub-themes were identified in the analysis, namely: Influences on breastfeeding beyond 1 year (family, friends, peers, culture, CMF marketing), Sustaining breastfeeding (support, determination, knowledge, bed-sharing, the impact of the Covid-19 pandemic) and Benefits of breastfeeding beyond 1 year (nutrition, emotional bond, parenting tool, protection). These themes are explored further below.

### Influences on breastfeeding beyond 1 year

A common theme identified in the interviews was the positive and negative influences on breastfeeding beyond 1 year. Among these influences were family, friends, peers, culture and CMF marketing.

#### Family, friends and peers

Familial influences on the women’s breastfeeding decisions and practices were reported to be very important. Family infant feeding practices and exposure to breastfeeding within families differed, with most women reporting that their mothers did not breastfeed. Nevertheless, many felt their family was a positive influence on their decision to breastfeed and during the first year of breastfeeding. However, in some instances, this changed as breastfeeding continued after the child’s first birthday:

My Mum who like breastfed kids, she definitely fed one or two beyond the year and maybe 13 or 14 months, but even then, she’s like ‘she’s grand so stop it’! [P6]

Friends were also identified as key influencers. There were differences in infant feeding practices within friendship groups. Some women reported that most of their friends had exclusively formula-fed their babies. Several women noted that some of their friends had breastfed their babies, mostly for a short period of time. Friends that had breastfed for beyond 1 year positively influenced the women to continue breastfeeding:

Maybe it’s the bubble that I live in, I don’t know I hope it’s not, but I have experienced quite a few friends that have breastfed long term. [P4]

The power of the peer relationship was evident in most of the women’s accounts of their experience of breastfeeding beyond 1 year. Exposure to other women breastfeeding was identified as an important influence. Several women observed other women breastfeeding older infants and toddlers at breastfeeding support groups. This helped normalize the practice and gave them encouragement to do so. One woman commented that most of the people she knew were ‘breastfeeders’ and went as far to say she surrounded herself with people who breastfeed beyond 1 year stating, ‘it is a real echo chamber I live in!’ [P1]

#### Culture

Women referred to a breastfeeding culture in the RoI where breastfeeding beyond 1 year ‘is not the norm- it’s far from the norm’ [P13]. The women perceived society’s view on breastfeeding a toddler as negative, which they believed was a barrier to continuing to breastfeed beyond a child’s first birthday:

I just think that culturally there’s such a negative association with it [breastfeeding beyond one year] that it does massively impact on the amount of people who do it for longer than six months, so it’s just total lack of education, like, for me, I didn’t even know you did feed after six months [P4]

One woman stated ‘there’s a pressure to give up from society’ [P5] and this is supported by another woman who stated that ‘anyone who succeeds at breastfeeding in Ireland succeeds despite major obstacles’ [P12]. Women reported that there was stigma and secrecy associated with breastfeeding beyond 1 year in the RoI. This led them to conceal the fact that they are still breastfeeding their toddler from family and friends:

And yeah, I do think there is a stigma around breastfeeding a larger baby, a baby that is walking around. [P8]

Oh yea, they [family] wouldn’t know that I am still feeding my son [P12]

This stigma and secrecy also influenced their breastfeeding practices in public. All women reported being comfortable breastfeeding an infant in public; however, this changed as their children grew older. Most women, both urban and rural dwelling, stated they were reluctant to breastfeed their toddler in public:

Like, he is now a big tall child. While I would definitely still feed him if he was upset or if he asked for it I would but like I would never offer it to him out. Because I would just feel like, I think I would feel that people are watching me. [P10]

Several women highlighted that the RoI is lagging behind other countries in terms of breastfeeding practices and culture. Knowing that it was the norm in other countries to continue breastfeeding into early childhood affirmed their choice and reinforced their belief in the importance of breastfeeding beyond 1 year:

Ireland is a kinda outlier, when you go outside of Ireland the rest of the world all sleep with their babies, the rest of the world naturally wean, you know children wean whenever they want you know- 3, 4 years of age. So we are actually the odd ones out- that gives me an element of comfort as well not that I care but I do often think that at least what I am doing is not completely mad! [P12]

Two women compared their experiences of breastfeeding in two different countries—the RoI and Australia. They both reported that Australia’s positive breastfeeding culture and environment facilitate breastfeeding and that this is in stark contrast to the RoI’s restrictive breastfeeding environment:

Their facilities are just phenomenal. You know they really facilitate breastfeeding and they have signs up everywhere you know ‘breastfeeding rooms here’, so many people breastfeed that no one bats an eye. [P14]

#### CMF marketing

Women identified the marketing of CMF as negatively influencing breastfeeding beyond 1 year in the RoI. They referred to various advertising channels used by CMF companies to target mothers and parents, including ‘pop ups’ on social media:

I would have seen an awful lot of pop ups coming up on my social media for [two names of CMF] and things like that, until I physically went and changed my settings so that I wouldn’t see them anymore. [P12]

The marketing of CMF such as follow-on products for ages 6–12 months and growing-up products for ages 13–36 months was viewed as subliminal. Several women reported how this aggressive marketing of CMF influenced their initial thinking which was to commence CMF after 6 months. When they became more informed, they had the confidence to continue breastfeeding and they avoided the introduction of CMF.

### Sustaining breastfeeding

Several factors were identified as being central to sustaining breastfeeding beyond 1 year. Support was crucial and came mainly from family, breastfeeding support groups and workplace colleagues. Deficits in support for breastfeeding beyond 1 year from healthcare professionals (HCPs) were also identified. Personal attributes such as determination and knowledge empowered the women to continue breastfeeding. Bed-sharing was identified by all the women as a facilitator for breastfeeding beyond 1 year. The social restrictions introduced during the Covid-19 pandemic also impacted the continuation of breastfeeding.

#### Familial support

Women reported a high level of support from partners ‘my husband is very supportive, he is a huge advocate [of breastfeeding beyond one year]’ [P12]. In particular, partners provided emotional support by encouraging the women to continue and providing reassurance through challenging periods:

he was very supportive and keep pushing me when I felt like I can’t do it anymore, or whatever like, he was ‘you can, you can, you can just one day at a time’. [P14]

he was the one who said ‘you don’t need to worry about this, this is absolutely fine, it is working’… having someone to just pat you on the shoulder when you are in that uncomfortable position and say no carry on. [P10]

We need to have the support of the baby’s dad 100% [P7]

The extended family was also identified as a significant support; however, in some cases, this was less evident as breastfeeding continued beyond 1 year. The decline of support was attributed to members of the extended family disapproving of breastfeeding beyond a year:

Everyone was very supportive like it was a really, really tough start like even my Mum commented afterwards, like that you know she couldn’t believe that I kept going, because it was obviously so difficult. I think, towards the end like maybe then sort of last six or eight months of when I was feeding her there was a few comments say from my dad he was like ‘oh she’s just doing it for comfort, or something like that’ [P11]

#### Breastfeeding support groups

The women accessed a range of breastfeeding support groups, including La Leche League and Public Health Nursing (PHN)-led groups which they found very supportive. These groups were traditionally face to face but they all facilitated online meetings during the Covid-19 pandemic.

All the women accessed one particular virtual support, a well-established Facebook page which supports breastfeeding beyond 1 year in the RoI. They unanimously found it very informative and a great support, giving them a sense of camaraderie—‘these are my people’ [P14]. They received information and guidance around a variety of issues relevant to breastfeeding beyond 1 year including returning to work and nursing strikes such as temporary or prolonged refusal of the breast:

It is an amazing resource- just knowing people are doing the same thing. If you are feeding children later on you are more likely that something is going to come up that you need to check it is ok. [P10]

It has been a fantastic group to be part of. I definitely would have put up a few questions and things like that along the way. [P8]

Online support targeted at very specific groups was also identified as being beneficial. Two women who were breastfeeding twins beyond 1 year received great support from members of a breastfeeding twins and triplets Facebook group:

Probably one of the biggest supports that I had over the whole time really. They have thousands of members but they like provided probably more advice than anyone else in terms of the breastfeeding journey because. I suppose it was quite specific around that kind of twin situation. [breastfeeding twins] [P5]

#### Workplace support

Following the lifting of the public health restrictions imposed during the Covid-19 pandemic, a number of the women returned to their workplaces having been office based prior to the pandemic. Workplace supports for breastfeeding beyond 1 year varied. It was apparent that management influenced support, in positive and negative ways. The level of support the women received was dependent on their individual line manager rather than any employee policies that were in place. Managers who had breastfed themselves or whose partner had breastfed were inclined to support women more than managers who had no exposure or experience of breastfeeding. Bespoke arrangements were common:

Everything comes down to your individual manager. It doesn’t really matter about the company or their policies or anything it comes down to individual managers being supportive or not. [P11]

The women’s physical workplace environments varied, with some reporting that they had to express breastmilk in totally unsuitable places such as showers and toilets. Other workplaces provided a dedicated breastfeeding room where mothers could express breastmilk during work thus positively influencing the continuation of breastfeeding beyond 1 year:

So there was a room with a fridge in there and a chair, where you could pump and the whole lot, and you could lock the door and there was a code to get in. [P14]

#### Deficits in support from HCPs

Professional breastfeeding supports were somewhat fragmented during the women’s early breastfeeding days due to the Covid-19 pandemic when home visiting by health professionals, face-to-face support group meetings and visits to health clinics were restricted.

Several women experienced a lack of support and understanding from health professionals when interacting with the health service as mothers who were breastfeeding a toddler. One woman was admitted unexpectedly to a general hospital due to a medical condition and as she was separated from her child, her breasts became engorged and she had to express breastmilk. She received no support from the HCPs who were caring for her despite being in significant and obvious distress:

I wasn’t even given anything to express into you know that’s why I ended up being in such a desperate position that I was expressing into the sink. I was in agony, but I found like there was no sympathy, no support, no acknowledgement to this whatsoever- a bit from the nurses, but not from the doctors [P7]

#### Determination

Most of the women reported experiencing breastfeeding challenges in their initial breastfeeding journey—pain and low milk supply being the most common difficulties reported. Despite the challenging start, they persisted and were determined to continue breastfeeding beyond their child’s first birthday. There was a sense of achievement in continuing to breastfeed beyond their child’s first birthday:

I found the start of the breastfeeding journey really difficult. Like really, really, really hard and eventually it started to get better around six months, and I was like oh I’ve worked so hard at this I’m not just jacking it in now. It is so much easier now, it’s been worth the effort! [P6]

Oh, I’m definitely aiming for the two years now…if I could hit the two years I’d be quite proud of myself. I am very determined when I want to be. [P9]

#### Knowledge

Many of the women reported lack of knowledge of breastfeeding beyond 1 year when they began breastfeeding their firstborn infant, with several revealing they were not even aware of the WHO recommendation of breastfeeding infants beyond 6 months: ‘I thought you only breastfed a baby for six months’ [P6]:

But like I remember asking the PHN, so you know so like is it cow’s milk or what do they get after 6 months? and she was like no, you keep breastfeeding with the food and I was like ‘oh’! It’s not clear, I was like I just assumed that was what happened. [P4]

While many women were poorly informed initially about breastfeeding, it was evident that their knowledge improved as their breastfeeding journey progressed. They became more informed through reading about breastfeeding and via informational support from peers. They learned more about the benefits of breastfeeding for mothers:

I knew all that information about how good it is for the babies before I started breastfeeding, but it’s only as I’ve become interested in it that I’ve realized in terms of kind of that it reduces your own chance of breast cancer and things like that I never quite realized that early on. [3]

Several women informed themselves about breastfeeding through reading books, and from peers:

My friend who was breastfeeding she gave me the Womanly Art of Breastfeeding. Yes, such an amazing book, like that book is so good [P2]

Because I had gone to so many groups and I heard so much information I realised at six months there is absolutely no need to stop breastfeeding like if I want to keep going like that’s fine and I went God Almighty why would I stop this this is fierce handy you know like it puts her back to sleep really quick, it is really handy sure why wouldn’t I just keep going. [P1]

#### Bed-sharing

All the women reported sharing a bed with their toddler, mainly to facilitate breastfeeding but also to reduce the disruption to their sleep. The nature of bed-sharing arrangements differed in terms of when the practice began. First-time mothers tended to delay bed-sharing until their infant was several months old, whereas women who were breastfeeding for the second time or more reported co-sleeping from the beginning with their most recent baby:

I bedshare. With [second baby] I bed-shared from Day 1. I think with [first baby] maybe we were a little more nervous the first time around and we had him in a basket beside the bed. [P10]

Women were aware of the safety concerns of bed-sharing and in some cases, this deterred them from the practice in the early days of their breastfeeding journey. Some women informed themselves about bed-sharing through reading resources from well-recognized institutions such as the National Institute for Health and Care Excellence (NICE) and the Lullaby Trust in the UK. Their newly acquired knowledge influenced their subsequent decisions to bedshare:

I co sleep and we’re still doing it most of the time. Initially I was very reluctant to because of the SIDS advice, but thankfully, I think because of the advice has been updated in the last year and a half around co sleeping or, at least from the Lullaby Trust in the UK, and yeah I felt like if the NICE guidelines in the UK, were finding it was safe I got rid of my anxiety around co sleeping, and also like I just when she wasn’t beside me I couldn’t sleep. when she was beside me, I could relax a little bit [P6]

Conflicting advice from HCPs around bed-sharing was common, and several women argued that parents require more balanced, evidence-based information on the practice, including advice on risk reduction strategies:

Like it’s just I think they need to let people know the safe ways to do it, rather than just say this isn’t a good idea full stop. [P5]

The two women who had twins bed shared and attributed the practice to facilitating breastfeeding twins beyond 1 year:

I co sleep and I still do co sleep with the twins. I think, like, most people who breastfeed twins will co sleep, it’s so hard to breastfeed otherwise I don’t know how you manage [P5]

#### Impact of the Covid-19 pandemic restrictions

All the women reported spending a lot of time at home with their babies due to the public health measures imposed during the Covid-19 pandemic. This helped sustain breastfeeding in the early days as there were no distractions, visitors to entertain or pressure to go out and about to meet people: ‘it was completely acceptable to actually just sit on the couch with my baby’ [P9]

Interestingly, several women reported that the restrictions facilitated them to continue breastfeeding beyond 1 year. Having the option to work from home allowed them to remain with their breastfeeding toddler rather than juggling commuting, being separated from their toddler and expressing breastmilk:

Covid changed all that like, it made like I said the ‘Buffet’ [breastfeeding] was more available [P11]

I was returning to work, from home, and that made it so much easier to facilitate breastfeeding [P6]

### Benefits of breastfeeding beyond 1 year

Many women referred to the benefits of breastfeeding beyond 1 year in their interviews. The benefits articulated included nutrition, the emotional bond with the infant or child, parenting the toddler and protecting both the mother and child.

#### Nutrition

Most of the women became aware of the WHO’s recommendation of breastfeeding for the first 2 years of life, and the associated nutritional benefits for the child. One dismissed the common view that there is little or no nutritional benefit of breastfeeding beyond 1 year:

The WHO guidelines- they say 2 [years] and beyond. I said I would really like to get to 2 and then beyond that I don’t care. [P10]

I don’t believe in this thing of like once they are over one [year] you know there’s no nutritional benefit…. I just I just don’t believe that because of course there is nutritional benefit [P11]

#### Emotional bond

The strength of the emotional bond that develops through breastfeeding beyond 1 year was very important for almost all the women. They noted that breastfeeding was not just about nutrition but also a connection with their child. So much so that interrupting this emotional bond by returning to work proved difficult for several women:

Knowing that he was all day without that comfort [breastfeeding] you know it’s hard like I found it really difficult to be honest and but you know it got easier [P2]

#### Parenting tool

Breastfeeding beyond 1 year was seen among other things as a great support to parenting. Parenting a toddler in the absence of such a tool was seen as very challenging. Breastfeeding was described by one woman as being ‘an awesome toddler tool- an everything tool’ [P3]. This was supported by several participants:

Like it’s free, and people don’t realize how helpful it is when they’re like having a tantrum, sore or upset. Yeah, amazing like it really is the most amazing parenting tool I have for sure, it just calms them down. Especially if he has hurt himself and when they’re sick [P4]

I just think it provides a soothing balm if there’s any kind of upset or anything in a way that not many other things can do [P11]

#### Protection

The women identified the health benefits of breastfeeding beyond 1 year and reported it as being protective for both the infant/child and the mother. At the time of the interviews, children under 5 years of age were not eligible to receive the Covid-19 vaccination. Several of the women believed that immunity to the virus was transmitted via breastmilk, so continuing to breastfeed their child was very important to them. Interestingly, several women reported the benefits through a public health lens and commented on the positive economic effects of breastfeeding for a significant length of time:

I personally don’t think of the benefits of breastfeeding- I think of the risks of not breastfeeding and I think the risk of not breastfeeding in terms of the mammy are very undersold in Ireland- you know your risk of cancer, diabetes are very undersold here, we don’t really talk about them at all. [P1]

You know if you look at the savings that are there long term to be made if people aren’t going to hospital for the childhood illnesses that you’re avoiding. But also things like breast cancer is you know the rates are reduced significantly if you breastfeed, for you know, a couple years out of your life does that makes a significant impact. [P5]

## DISCUSSION

This qualitative study was one of the first to explore women’s experiences of breastfeeding beyond 1 year in the RoI. Given the known benefits of breastfeeding beyond 1 year, it is important to explore the practice in the context of the RoI where breastfeeding rates are very low. The women in our study were determined, had a high degree of self-efficacy and, as they embraced their breastfeeding journey, became more confident in the art of breastfeeding over time.

The importance of family, friends, peers and culture as influences on breastfeeding beyond 1 year is demonstrated in this study. Breastfeeding support groups and clinics provided, for the first time, the visual realization that breastfeeding beyond the first few months of life was normal, suggestive of social learning theory behaviours (Bandura, 1977). Indeed, this has been alluded to in previous research studies in the RoI where mothers reported the value of sharing breastfeeding experiences and parenting information with each other (Leahy-Warren *et al*., 2017; McCarthy Quinn *et al*., 2019; McGuinness *et al*., 2020).

Although all the women were highly educated, the analysis revealed they initially had suboptimal health literacy in relation to breastfeeding, particularly breastfeeding beyond 1 year. However, their knowledge and competence improved as their breastfeeding journeys progressed. Several women commenced breastfeeding with the incorrect belief they should wean their infant from the breast at 6 months of age. They attributed this belief to the intense marketing of CMF through social media such as ‘pop ups’ which appeared as they searched for infant feeding content during the perinatal period. This demonstrates how commercial companies have the potential to influence breastfeeding outcomes and supports the growing concern and complexities around the Commercial Determinants of Health (CDoH) (McCarthy *et al*., 2023). Such advertisement and promotion of CMF portrays it as a normal and acceptable food for infants (Philip *et al*., 2023).

While there are negative influences of social media on breastfeeding initiation and duration such as through advertisement of CMF as outlined above, the benefits of social media are well documented. Morse and Brown (2022) in their systematic review note the positive impact of breastfeeding support via social media and report that women value the accessible nature of online support and belonging to a community that normalizes breastfeeding. In addition, support from social media breastfeeding groups was linked to an increase in duration of breastfeeding. This was echoed in the findings of this current study in which all the participants were active members of online support groups which they found invaluable.

Support from peers around breastfeeding beyond 1 year was crucial. Peer support was received from face-to-face breastfeeding support groups as well as via online support groups, particularly during the pandemic. As previously outlined by Chang *et al.* (2022), peer support increased the women’s confidence in breastfeeding, provided practical and emotional support and camaraderie. Jackson and Hallam (2020) reported online peer support as being particularly helpful in providing information on breastfeeding beyond 1 year and facilitating support at any time, with some participants in their study reporting that they would seek advice from their peers before talking to HCPs.

Workplace support for breastfeeding was also highly valued by the women interviewed. Some managers were aware of the importance of a designated breastfeeding room for breastfeeding women returning from maternity leave. Recently enforced workplace legislation in the RoI entitles breastfeeding women in the workforce to breastfeeding breaks, in addition to normal rest breaks, until the child’s second birthday (Government of Ireland, 2023).

The women in the study had experienced breastfeeding during the Covid-19 pandemic. Most of the women associated the public health restrictions as aiding the continuation of breastfeeding beyond 1 year. Similar to recent research by Turner *et al.* (2023) and Chien *et al.* (2022) on breastfeeding during the pandemic, the women had more time to focus on their baby with less visitors and pressure to meet people during the early days of breastfeeding. They reported that the restrictions facilitated the sustaining of breastfeeding for longer than they anticipated.

Thompson *et al.* (2020) reports that women may be reluctant to seek advice from HCPs as they are perceived to lack evidence-based knowledge, and for fear of negative responses and opinions. This reflects Baranowska *et al*.’s (2018) finding that HCPs had insufficient knowledge about breastfeeding beyond 1 year, which was also the experience of some of the women in this study. The focus of HCPs breastfeeding education tends to be on supporting the initiation of breastfeeding and early breastfeeding practices. A recent study conducted in the RoI identified significant gaps in GPs and Practice Nurses knowledge of breastfeeding in general and that there was a need for specific training in lactation for this cohort (McGuinness *et al*., 2024). Given the findings of the current study, it is vital that the curriculum for such training includes content on breastfeeding beyond 1 year.

The current study highlighted stigma and a negative breastfeeding culture in Ireland, particularly in relation to breastfeeding a toddler or older child. This aligns with research from other jurisdictions such as Jackson and Hallam’s (2021) study in which women reported an element of stigma associated with breastfeeding beyond a year to the extent that they concealed it from their family and friends. Over three decades ago, the (WHO, 1987), through the Ottawa Charter, argued the importance of creating supportive environments for health. This is reflected in Leahy-Warren *et al*.’s (2017) call for the need for a ‘cocoon’ of normality for breastfeeding women in the RoI, promoting a culture where embarrassment and negative attitudes are removed. Grant *et al.* (2022) and Grant (2021) highlight the need to change ‘hostile’ beliefs and environments that present barriers to breastfeeding in public. Increasing the visibility of breastfeeding, education of the general public and a breastfeeding-friendly environment are all factors identified by Irish, Swedish and Australian women as interventions that could assist in the promotion of a positive breastfeeding culture (Dykes *et al*., 2023).

Night-time breastfeeding practices were highlighted by the women in the study. Current advice from some national organizations supports a safe sleep environment to reduce the risk of SIDS with one key recommendation being to separate the infant from the parent’s bed (Moon *et al*., 2022). In this current study, all the women practiced bed-sharing to sustain and prolong breastfeeding, a reason previously identified by Ball *et al.* (2016). Several reported how their sleeping arrangements changed over time—due to concerns around SIDS, they avoided bed-sharing from birth with their first baby—but contrary to national guidelines in the RoI (Health Service Executive, 2024), they engaged in bed-sharing with subsequent babies. The women were well informed and referred to guidelines from the UK such as the NICE (2021) which no longer advises against bed-sharing in the absence of hazardous circumstances such as tobacco, drugs and alcohol. In addition to prolonging the duration of breastfeeding, bed-sharing improved the women’s sleep. This is of importance as poor sleep quality has been associated with maternal postpartum depression and anxiety (Okun *et al*., 2018), and breastfeeding has been associated with a reduced risk of postpartum depression (Alimi *et al*., 2022).

While the early breastfeeding journey was associated with the importance of breastfeeding for child and maternal health, the women progressed to using it as a ‘parenting tool’. Breastfeeding beyond a year was associated with strong mothering instincts—attachment parenting, emotional bonding, a connection, comfort and support for infant sleep. This parenting style instinctively developed as breastfeeding continued for the mother/infant dyad, consistent with findings from Thompson *et al.* (2020). Research has described infant attachment security among infants predominantly breastfed for 6 months or longer (Gibbs *et al*. 2018). This view is supported by Jackson and Hallam (2021) who identified that breastfeeding could enable women to play a key role in their child’s health with an attachment led parenting style evolving. Attachment parenting is described as a style that promotes a strong emotional and physical connection to one main care provider (Thompson *et al*. 2020).

### Limitations of the study

The sample in this study was homogenous: women aged in their 30’s and 40’s, with a high level of education, in a heterosexual relationship and married or cohabiting with their partner. Trans and non-binary people were not represented in the sample. The demographics of the participants align with those more likely to breastfeed for longer in the RoI, as it has been documented that breastfeeding rates in this setting are significantly associated with increasing maternal age, higher social class and higher levels of education (McCrory and Layte, 2014). Future research, possibly of a quantitative design, would be beneficial to explore the issue further and could examine the practice of breastfeeding beyond 1 year among a broader population.

## CONCLUSION

This study reports women’s experiences of breastfeeding beyond 1 year in the RoI, highlighting the benefits for the breastfeeding dyad. Women in the study became confident in the art of breastfeeding over time, overcoming challenges along the way. Support from an online breastfeeding community was an invaluable facilitator in sustaining breastfeeding beyond 1 year. Within the current trend to support breastfeeding dyads, our findings support discussions and further research on the influence of social media on long-term breastfeeding and mechanisms of online support for sustaining breastfeeding; the normalization of breastfeeding beyond a year; the importance of health promotion initiatives for the general population; and targeted education programmes for HCPs on supporting the breastfeeding dyad beyond infancy.

## Supplementary Material

daae088_suppl_Supplementary_Files_1

## Data Availability

The data underlying this article will be shared on reasonable request to the corresponding author.
