# Supplementary material for: ‘It’s far from the norm’: breastfeeding beyond 1 year in the Republic of Ireland
Source: Health Promot Int. 2024 Aug 17;39(4):daae088. doi: 10.1093/heapro/daae088 (PMC11329779; doi:10.1093/heapro/daae088)
Supplement: daae088_suppl_Supplementary_Files_1 [file daae088_suppl_supplementary_files_1.docx]

**SUPPLEMENTARY FILE**

***“It’s far from the norm”*: breastfeeding beyond one year in the Republic of Ireland**

**Semi structured interview guide**

The following is an outline of the questions asked during the semi structured interview:

- How old is your baby (that you are currently breastfeeding)?
- How long have you been breastfeeding this baby?
- Recalling your initial decision to breastfeed your baby:
  - Can you tell me about when you decided to breastfeed?
  - And why you choose to breastfeed?
- Tell me about your current breastfeeding journey:
  - What informed your decision to continue to breastfeed beyond your baby’s first birthday?
  - What has helped you to breastfeed for this long?
    - Can you explain in more detail?
  - Have you experienced any barriers to continuing breastfeeding beyond one year?
    - If so can you describe them to me?
    - Can you explain how you overcame them?
  - Where have you sought information and support on breastfeeding beyond one year?
    - Were you happy with the information and support you received?
    - If not, why?
- What has been your experience of interacting with the health service and health care professionals in relation to breastfeeding beyond one year?
- Have you been in a position where you had to return to work whilst continuing to feed your baby?
  - If so, how has this experience been for you?
  - Did you encounter any difficulties?
    - Could you tell me more about these?
- Would you like to tell me anything further about your breastfeeding experience?
